# Müllerian-Type Clear Cell Carcinoma of Donor Origin in a Male Patient with a Kidney Transplant: Ascertained by Molecular Testing

**DOI:** 10.3390/curroncol30100651

**Published:** 2023-10-05

**Authors:** J. Bryan Iorgulescu, Leah K. Shaw, Asif Rashid, Priya Rao, Sreedhar Mandayam, Keyur P. Patel, Kathleen M. Schmeler, Richard K. Yang, Pavlos Msaouel

**Affiliations:** 1 Molecular Diagnostics Laboratory, Department of Hematopathology, Division of Pathology and Laboratory Medicine, The University of Texas MD Anderson Cancer Center, Houston, TX 77030, USA; arashid@mdanderson.org (A.R.); kppatel@mdanderson.org (K.P.P.); rkyang@mdanderson.org (R.K.Y.); 2 Department of Genitourinary Medical Oncology, Division of Cancer Medicine, The University of Texas MD Anderson Cancer Center, Houston, TX 77030, USA; lkshaw@mdanderson.org; 3 Department of Pathology, Division of Pathology and Laboratory Medicine, The University of Texas MD Anderson Cancer Center, Houston, TX 77030, USA; prao@mdanderson.org; 4 Department of Section of Nephrology, Division of Internal Medicine, The University of Texas MD Anderson Cancer Center, Houston, TX 77030, USA; samandayam@mdanderson.org; 5 Department of Gynecologic Oncology and Reproductive Medicine, Division of Surgery, The University of Texas MD Anderson Cancer Center, Houston, TX 77030, USA; kschmele@mdanderson.org; 6 Department of Translational Molecular Pathology, Division of Pathology and Laboratory Medicine, The University of Texas MD Anderson Cancer Center, Houston, TX 77030, USA; 7 David H. Koch Center for Applied Research of Genitourinary Cancers, The University of Texas MD Anderson Cancer Center, Houston, TX 77030, USA

**Keywords:** clear cell carcinoma, Müllerian type, transplant kidney, short tandem repeat testing, next-generation sequencing

## Abstract

Clear cell carcinomas of Müllerian origin have a strong female predominance and only extremely rarely will arise within the kidney, presumably due to ectopic Müllerian embryogenesis. Herein, we report a unique case of metastatic Müllerian type clear cell carcinoma in a 37-year-old patient who had previously received a transplanted kidney from his father at age 11 (due to severe bilateral vesicoureteral reflux) and remained on chronic immunosuppression. The tumor was highly aggressive and demonstrated somatic mutations in *NF2* and *SETD2.* Imaging of the transplanted kidney did not reveal any clear evidence of malignancy. However, targeted multigene sequencing and short tandem repeat testing revealed that the cancer was of donor origin, presumably from ectopic Müllerian tissue transplanted to the patient along with the kidney graft. The tumor was resistant to first-line therapy with a triple combination of carboplatin plus paclitaxel plus bevacizumab, as well as to second-line immunotherapy with nivolumab plus ipilimumab after tapering down the patient’s immunosuppression. Despite the tumor being genetically distinct from the host, the use of immune checkpoint therapy with nivolumab plus ipilimumab did not yield a response. This unique case showcases the value of molecular testing in determining the tumor origin in patients with solid organ transplants who present with cancers of unknown primary. This can prompt the potential investigation of other recipients from the same donor.

## 1. Introduction

Clear cell carcinomas (CCCs) of the Müllerian system are relatively rare entities that predominantly arise in the female genital tissues (endometrium, ovary, uterine cervix, and vagina), affecting women and girls of all age groups [1,2,3]. Conversely, CCC of the Müllerian type very rarely arises in the male genitourinary (GU) tract, presumably due to ectopic Müllerian embryogenesis [4]. In the majority of such cases, the CCC occurs in the bladder and urethra, whereas CCC of the upper urinary tract is exceedingly rare, with less than five cases reported to date [5]. Although a case of conventional urothelial carcinoma with divergent Müllerian-type CCC differentiation arising from the renal pelvis of a kidney graft in a male patient was recently reported [6], CCC of the Müllerian type arising from transplanted organs has not been described to date. Herein, we report a unique case of metastatic Müllerian-type CCC in a young male patient arising from a kidney transplant of paternal origin. Although the tumor was initially misclassified at the external hospital as clear cell renal cell carcinoma (RCC), a comprehensive work-up facilitated the correct diagnosis and tailored management of this rare and aggressive histology.

## 2. Case Presentation

### 2.1. Initial Clinical Presentation

A 37-year-old male presented with a 4-day history of right lower abdominal pain, nausea, and vomiting. His past medical history was significant for hypertension and genitourinary dysplasia. He consequently developed vesicoureteral reflux, eventually nephropathy, for which he underwent left nephrectomy at the age of 1 year. Over the subsequent years, the vesicoureteral reflux gradually led to right kidney atrophy, necessitating hemodialysis. At the age of 20 years old, he subsequently underwent a living, related kidney transplant in the right pelvis donated by his father. After the kidney transplant, he remained on chronic immunosuppression with stable doses of tacrolimus 1.5 mg per os (PO) twice daily, prednisone 5 mg PO daily, and azathioprine 150 mg PO daily.

### 2.2. Diagnostic Imaging

**Figure 1:**
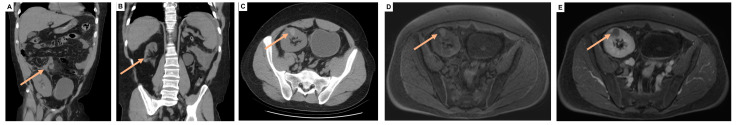
(**A**) Coronal CT imaging showing acute appendicitis (arrow) at presentation. (**B**) Coronal CT view of the native right atrophic kidney (arrow). (**C**) Axial CT view of the right pelvic kidney transplant, which harbored cysts of varying densities (arrow). (**D**,**E**) T1-weighted MRI view of the right pelvic kidney transplant, which demonstrated T1 hyperintense signal (**D**) that did not enhance with gadolinium contrast (**E**) and was thus suggestive of a proteinaceous/hemorrhagic cyst.

Computed tomography (CT) imaging performed at the time of presentation in the emergency room was consistent with acute appendicitis with prominent retroperitoneal and pelvic lymph nodes (Figure 1A). The imaging also revealed an empty left renal fossa, an atrophic right native kidney, and a transplanted kidney with multiple cysts of varying densities that were otherwise unremarkable, with no evidence of malignancy (Figure 1B,C).

### 2.3. Surgery and Histopathologic Work-Up

**Figure 2:**
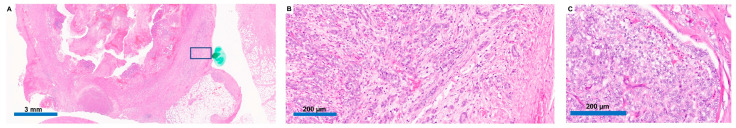
(**A**) Histological evaluation via hematoxylin and eosin staining showed a peri-appendiceal lesion, with the area in the blue rectangle magnified in (**B**) and revealing an unusual heterogeneous neoplasm composed of solid and tubular components with clear cell morphology. (**C**) The peritoneal lesion showed a similar morphology.

The patient subsequently underwent appendectomy. Omental nodules were noted, which were biopsied during the surgery. Pathology showed acute appendicitis and perforation, as well as an unusual peri-appendiceal heterogeneous neoplasm that was composed of both solid and tubular components with clear cell morphology (Figure 2A,B). The biopsied peritoneal nodules showed a similar morphology (Figure 2C).

**Figure 3:**
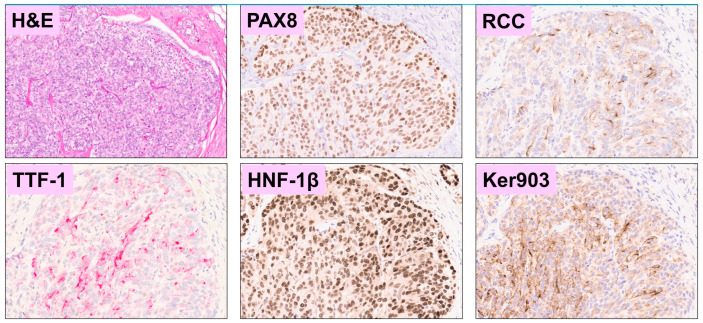
Immunohistochemical staining of the patient’s tumor was positive for PAX8, RCC, HNF-1β, and cytokeratin 903 and negative for TTF-1, with the corresponding hematoxylin and eosin-stained section for the same region of interest. All panels are at 100× magnification.

Initial pathology evaluation at the community center where the appendectomy took place diagnosed the tumor as a clear cell RCC. However, the morphological features were unusual for clear cell RCC, and subsequent immunohistochemical (IHC) staining at our institution was consistent with Müllerian-type CCC (Table 1) [7,8,9]. Notably, there was positive staining for Napsin-A, P504S (AMACR), and HNF-1β (Figure 3).

Biopsy of the transplanted kidney was negative for malignancy and was consistent with longstanding hypertensive nephrosclerosis, with no evidence of transplant glomerulopathy or thrombotic microangiopathy on light and electron microscopy, although peritubular capillaries demonstrated focal mild increases in peritubular capillary basement membrane layers (up to 5 layers). There was a single glomerulus with segmental sclerosis, while podocyte foot processes were generally preserved. There was no evidence of active rejection; however, arterial and arteriolar nephrosclerosis were noted with 38% global glomerulosclerosis (12/32 glomeruli in all specimens) and 10% interstitial fibrosis with tubular atrophy.

A diagnosis of Müllerian-type CCC (possibly arising from the peritoneum) was tentatively made. An enlarged right pelvic lymph node was subsequently biopsied and showed the same malignant histologic features as the peri-appendiceal and omental lesions. IHC for PD-L1 (using the 22C3 antibody) of the metastatic lymph node biopsy showed the tumor cells to be non-immunoreactive. Tumor-associated lymphocytes and macrophages were also negative for PD-L1 expression; a combined positive score was approximately zero. Serum alpha-fetoprotein (AFP) was 8.3 ng/mL, β-human chorionic gonadotropin (β-HCG) was 1.2 mIU/mL, lactate dehydrogenase (LDH) was 166 U/L, CA 19-9 was 5.6 U/mL, and carcinoembryonic antigen (CEA) was undetectable, while CA 15-3 was slightly elevated at 39.4 U/mL, and CA-125 was substantially elevated at 228 U/mL.

### 2.4. Molecular Testing Results

Germline testing (Invitae) was negative for germline pathogenic variants in any of the following analyzed genes: *ATM*, *BAP1*, *BRCA1*, *BRCA2*, *CDH1*, *CHEK2, EPCAM*, *FH*, *FLCN*, *MET*, *MITF, MLH1*, *MSH2*, *MSH6*, *PALB2*, *PMS2*, *PTEN*, *SDHB*, *SDHC*, *SDHD*, *STK11*, *TP53*, *TSC1*, *TSC2*, and *VHL*.

**Figure 4:**
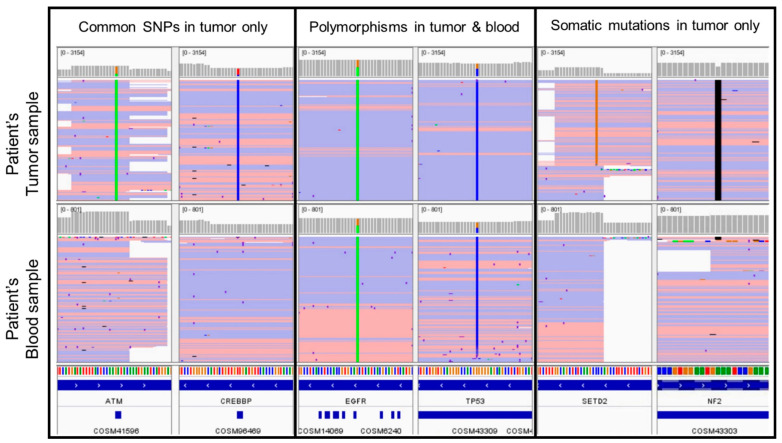
NGS-based targeted sequencing analysis of tumor tissue (**top**) and peripheral blood (**bottom**) obtained from the patient displaying representative (1) common SNPs that were detected in the patient’s tumor (but not blood) sample (**left**), (2) SNPs detected in both the patient’s tumor and blood samples (**middle**), and (3) somatic mutations detected in the patient’s tumor—(but not blood) sample (**right**). SNP: single nucleotide polymorphism.

Matched tumor-normal (peripheral blood) DNA sequencing targeting 134 genes via polymerase chain reaction (PCR)-amplicon-based target capture next generation sequencing (NGS) [10] of the peritoneal metastasis biopsy, and subsequent lymph node metastasis biopsies identified an *NF2* frameshift deletion and *SETD2* missense single nucleotide variant (Table 2), as well as no gene fusions. This was the largest panel available at our institution at that time and included evaluation of amplifications in 47 genes (in which none were detected). Of note, neither *VHL* nor an assessment of copy number losses were included in the panel. Several polymorphisms were identified in both the patient’s tumor and blood samples at near heterozygous variant allele frequencies (VAFs). In addition, at least 7 variants (with VAFs similar to the *NF2* and *SETD2* mutations) were identified in the tumor samples, which are well-characterized single nucleotide polymorphisms (SNP) frequently present in population databases, such as the Single Nucleotide Polymorphism Database (dbSNP). However, these variants were not identified in the patient’s matched peripheral blood samples (Figure 4).

However, these SNPs were identified in sequencing of the donor’s blood. Subsequent PCR-based short tandem repeat (STR) testing using 24 loci of the patient’s tumor sample, the patient’s peripheral blood, and the donor’s peripheral blood demonstrated that: (1) 11/12 informative markers detected in the patient’s tumor were also identified in the patient’s peripheral blood, and (2) 12/12 informative markers detected in the patient’s tumor were also identified in the donor’s peripheral blood. Overall, the alleles detected in the patient’s tumor sample comprised a mixture of the patient and the donor. Therefore, the targeted matched tumor-normal sequencing and STR testing of this metastatic cancer of unknown primary traced the origin to the patient’s kidney transplant. This established the final diagnosis of metastatic Müllerian-type CCC of kidney transplant origin.

### 2.5. Management and Outcomes

Re-staging imaging one month after the appendectomy revealed widely metastatic retroperitoneal and pelvic lymphadenopathy with peritoneal carcinomatosis and ascites. The patient was subsequently started on cytotoxic chemotherapy with carboplatin AUC 5, paclitaxel 175 mg/m^2^, and bevacizumab 15 mg/kg every 3 weeks, a regimen known to be effective in advanced ovarian CCC [11]. However, the tumor quickly progressed after 3 cycles of this therapy. His azathioprine was tapered off, and he was subsequently started on nivolumab 3 mg/kg in combination with ipilimumab 1 mg/kg every 3 weeks. The patient received two cycles of this immunotherapy and succumbed to his disease within 6 months of the diagnosis.

## 3. Discussion

Intraperitoneal Müllerian-type CCC is an especially rare malignancy, which often requires advanced diagnostic techniques to make the diagnosis. Our patient represents the first reported case of Müllerian-type CCC of donor origin in a patient with a kidney transplant, as evidenced by STR and NGS-based testing of the patient and donor samples. We hypothesize that the tumor likely originated from ectopic Müllerian tissue transplanted along with the kidney transplant (e.g., the renal pelvis), which showed no radiographic or biopsy evidence of malignancy. Post-mortem pathological evaluation of the kidney transplant was not conducted, and there remains the possibility of an occult primary source that was not detected via imaging.

Thus, the paternal donor origin of the tumor could only be identified through molecular testing. To ensure that no contamination could have occurred (given the proximity of the transplanted pelvic kidney to the patient’s appendix), the tumor NGS was performed in an omental nodule and a distant lymph node metastasis, both of which showed alleles that were a mixture of the patient and the donor, consistent with a donor origin for the metastatic malignancy. The presence of two different SNP profiles in the tumor tissue suggests that the tumor cells were of donor origin, whereas the non-neoplastic cells were of host origin.

Overall, genitourinary carcinomas originating from kidney allografts are rare. A retrospective study of 3568 renal transplants over more than four decades noted that only eight patients (0.2%) developed RCC, with five cases being papillary RCC, whereas three cases were clear cell RCC [12]. Conversely, 39 of the 3568 patients (1.1%) developed RCCs in their native kidneys [12]. Another retrospective analysis of 1584 renal transplant patients found that only 4 patients (0.3%) developed a GU malignancy in their allograft kidneys, with one case being clear cell RCC, one case being papillary RCC, one case being *TFE3* translocation RCC, and one case being urothelial carcinoma arising from the allograft pelvis at 6 years following BK virus infection [13]. In the present case, a BK virus assessment was not performed. A third retrospective study used STR analysis to discriminate between host and donor origins of RCC identified in renal allografts and found that 5 out of 542 renal transplants (0.9%) developed RCC, with histology being clear cell RCC in three cases, papillary RCC in one case, and RCC with oncocytic features in one case [14].

No prior study has noted the emergence of Müllerian-type CCC originating from a donor in a transplant patient, and this should be considered in the differential of patients with organ transplants who develop Müllerian-type CCC of unknown origin. Our case also highlighted the value of both NGS-based sequencing and STR analyses in determining the tumor origin in patients with solid organ transplants, particularly those whose primary tumor is not evident on imaging. For a sex-mismatched donor, X/Y chromosome fluorescence in situ hybridization can also be of help. Distinguishing between donor and host origin is important because it may prompt the potential evaluation of other organ recipients from the same donor. In the present case, the patient was the only recipient of a transplant from his father. Additionally, the tumor origin has implications for staging; for instance, a donor-origin RCC arising in a transplanted kidney may behave as stage I–II disease, but a host-origin RCC arising in a transplanted kidney would represent stage IV disease.

Although the histological and immunohistochemical findings supported the diagnosis of a Müllerian-type CCC, these features can also be observed in other rare cancer types, particularly in the setting of our molecular findings. In particular, the *SETD2* and *NF2* somatic mutations are not commonly reported in Müllerian-type CCC [15,16], which is frequently characterized by mutations in *ARID1A, PIK3CA, TP53*, and *PTEN* [17]. Additionally, our limited copy number evaluation did not reveal features associated with the *TP53* mutant (a surrogate for high copy number) or specific molecular profile (a surrogate for low copy number) subgroups defined by The Cancer Genome Atlas classifier of endometrial carcinomas and Müllerian-type CCC [17,18].

Together, the molecular profile raised the concern that this case may be an RCC. However, whereas *SETD2* mutations are commonly observed in clear cell RCC, *NF2* mutations are found in only 0.9–3% of clear cell RCC cases [19,20]. Furthermore, the recently described *NF2*-mutated unclassified RCC variants do not demonstrate a clear cell morphology [21]. Even if this was indeed an RCC or another less common cancer, the present case underscores the importance of molecular testing in identifying the origin of the tumor in patients who have received solid organ transplants and have subsequently developed cancers of unknown primary with no tumor radiologically evident in the transplant organ.

By the time the diagnosis of Müllerian-type CCC was made, the malignancy was already well advanced. Müllerian-type CCC is known to be highly aggressive, and a poor outcome often occurs once the tumor is disseminated. For instance, the patient’s tumor was refractory to the standard triplet cytotoxic chemotherapy regimen of carboplatin, paclitaxel, and bevacizumab used in advanced ovarian CCC [11]. Furthermore, the tumor tissue was negative for PD-L1 expression, and combination immune checkpoint therapy with nivolumab plus ipilimumab did not produce a response, despite the tapering down of immunosuppression by discontinuing azathioprine while continuing low-dose tacrolimus and prednisone. In patients with kidney transplants, the use of nivolumab plus ipilimumab may increase the risk of acute rejection due to increased T-cell responses [22]. Rejection typically occurs within 2-3 weeks from immune checkpoint therapy initiation and is associated with high mortality rates [16]. Our patient did not show any evidence of graft rejection for more than 9 weeks after immune checkpoint therapy initiation. Furthermore, a multicenter, single-arm, phase 1 study in patients with kidney transplants found that maintaining baseline immunosuppression may not affect anti-tumor efficacy [23]. Depending on the extent of homology of HLA alleles between patient and father, the tumor-specific donor SNPs could theoretically increase the chances of immune recognition of tumor cells following nivolumab plus ipilimumab. However, the lack of response of our patient’s Müllerian-type CCC to the strongest currently available immune checkpoint therapy combination suggests that novel immunotherapy or other therapeutics are needed for this rare (but aggressive) histology. Furthermore, molecular profiling is emerging as an important tool for helping ascertain what may be the best approach for choosing chemotherapeutic and/or immunotherapeutic agents.

## 4. Conclusions

Taken together, our case underscores that in NGS-based paired tumor-normal sequencing, the presence of well-characterized SNPs that are exclusive to the tumor specimen should include a transplant donor origin in the differential (in addition to the potential for a sample swap involving the normal specimen). STR/microsatellite testing of the patient and donor can help confirm the origin in such instances.

## Figures and Tables

**Table 1:** Immunohistochemical markers tested on tumor cells.

Positive Markers	Negative Markers
PAX8 RCC Cytokeratin 903 CD10 HNF-1β Napsin-A P504S BAP1 (intact)	TTF-1 GATA3 Cytokeratin 5/6 Cytokeratin 20 Cytokeratin 7 p63 S100 OCT4 PAX2 Mesothelin Calretinin CDX-2 PLAP

**Table 2:** Molecular analysis of tumor tissue and peripheral blood obtained from the patient and the transplant donor (patient’s father).

	Variant Allele Frequency (VAF)			
	Peritoneal Metastasis	Lymph Node Metastasis	Patient Blood	Transplant Donor Blood
**Somatic mutations in tumor only**				
*NF2* c.781del p.I261fs	8%	28%	0%	0%
*SETD2* c.4687G > C p.G1563R	13%	31%	0%	0%
**SNPs in tumor and patient’s blood**				
Multiple SNPs	~50%	~50%	~50%	0%
**Common SNPs in tumor but not patient’s blood**				
7 SNPs	8–10%	20–34%	0%	~50%

SNPs: single nucleotide polymorphisms.

## Data Availability

Data sharing not applicable. No new data were created or analyzed in this study.
